# Systemic Inflammatory Response Index (SIRI) Among Patients With Sepsis: A Retrospective Observational Study

**DOI:** 10.7759/cureus.104047

**Published:** 2026-02-22

**Authors:** Shubhransu Patro, Sidharth S Pattnaik, Parmarth Arora, Arushi Choudhary, Vibha Sharma, Prithviraj U Naik, Apoorav Mahajan, Yallambhotla Varuneil

**Affiliations:** 1 General Medicine, Kalinga Institute of Medical Sciences, Bhubaneswar, IND

**Keywords:** correlation analysis, healthcare associated infection, lymphocyte count, microcirculation and inflammation, monocyte count, neutrophil count, sequential organ failure assessment (sofa), severe sepsis, systemic inflammatory response index (siri), total leukocyte count

## Abstract

Background and objectives: Worldwide, sepsis and multiorgan dysfunction syndrome (MODS) are major health issues. The Sequential Organ Failure Assessment (SOFA) score is used to assess the severity of illness in patients with life-threatening conditions. A new composite biomarker for sepsis is the Systemic Inflammatory Response Index (SIRI). It is based on three metrics: absolute neutrophil count (ANC), absolute lymphocyte count (ALC), and absolute monocyte count (AMC). The aim of this study was to analyze and evaluate the SIRI of sepsis patients of both sexes. We also checked the association between the subjects' SIRI and SOFA scores at admission.

Methods: This study was conducted retrospectively at the Kalinga Institute of Medical Sciences (KIMS), Bhubaneswar, India, between August 2024 and July 2025. This study comprised adult patients of both sexes who met Sepsis-3 criteria. To calculate SIRI, we documented their ANC, ALC, and AMC. On days 1, 3, and 7, we calculated their SOFA scores. The Wilcoxon and chi-square tests were used to evaluate the continuous and categorical variables, respectively. To examine the association between the individuals' SIRI and their SOFA score on the first day, we employed the Spearman correlation. For data analysis, R software (version 4.3.2, R Foundation for Statistical Computing, Vienna, Austria) was utilized.

Results: A total of 2008 patients were analyzed in this study. Their median age was 61.0 (53.0-68.0) years. There were 1170 (58.27%) males. The average hospital stay was 11.0 (9.0-15.0) days, and 1569 (78.14%) subjects had MODS. The median ANC values for female and male subjects were 12.73 (10.41-15.28) x 10^9^/L and 12.42 (10.22-15.95) x 10^9^/L, respectively. The median ALCs of female and male subjects were 1.16 (0.79-1.83) x 10^9^/L and 1.29 (0.83-1.91) x 10^9^/L, respectively. The median AMCs of female and male subjects were 0.49 (0.33-0.71) x 10^9^/L and 0.59 (0.40-0.80) x 10^9^/L. The median SIRI values of female and male subjects were 5.11 (4.03-6.29) x 10^9^/L and 5.55 (4.26-6.88) x 10^9^/L. The median SOFA scores for female and male subjects at admission were 4.0 (2.0-6.0) and 5.0 (3.0-8.0), respectively. There was a negative correlation between SOFA score at day 1 and SIRI (r = -0.078, p < 0.001).

Conclusions: The ANC values were above the normal limits. But the ALC and AMC values were within the normal range. The hematological parameters of the male and female subjects were comparable. Male participants, however, had higher SIRI and SOFA scores. The SOFA score and the SIRI had a weakly negative association. A single-centric study design, a small sample size, and missing information on antibiotic use, daily fluid consumption, urine output, concomitant medications, comorbidities, and renal, hepatic, diabetic, and hemodynamic parameters prevent our study findings from being generalized.

## Introduction

Sepsis, a dysregulated host reaction to infection that results in organ failure, is an important cause of morbidity and mortality worldwide [1-3]. Sepsis and septic shock are severe systemic inflammatory reactions to infection [1,4]. A recent study found that the age-adjusted death rate per one lakh population was 111.8, 46.7, and 52.0 for pulmonary, abdominal, and genitourinary sepsis, respectively [5]. The mortality of sepsis patients is linked to abrupt failure of two or more organ systems brought on by sepsis [6,7]. Cytokines, such as interleukin-1beta (IL-1β), interleukin-6 (IL-6), and tumor necrosis factor-alpha (TNF-α), facilitate the generation of reactive oxygen (ROS) and nitrogen (RNS) species and alter mitochondrial permeability [1,6,8]. Both ROS and RNS are produced by immunological cells, such as neutrophils, monocytes, lymphocytes, and macrophages [8,9]. Elevated oxidative stress leads to mitochondrial dysfunction, which, in turn, causes cellular energy failure, cytopathic hypoxia, and cell death, all of which contribute to organ damage [6,10].

Critically ill patients were evaluated using the Sequential Organ Failure Assessment (SOFA) score [11]. Newer biomarkers for sepsis include intercellular adhesion molecule 1 (ICAM-1), procalcitonin, interferons, adrenomedullin, IL-6, and C-reactive protein (CRP) [12,13]. These biomarkers are more expensive and less available [12,14].

The Systemic Inflammatory Response Index (SIRI) is a new composite biomarker for sepsis [15,16]. It is based on three parameters: absolute neutrophil count (ANC), absolute lymphocyte count (ALC), and absolute monocyte count (AMC) [15,16]. SIRI is calculated using the following formula: ANC × AMC/ALC (unit: 10^9^/L) [15-18]. This indicator dynamically assesses immunological balance and systemic inflammation. Because of their active status, neutrophils, which are important for innate immunity, significantly influence the intensity of inflammatory responses [19]. Monocytes are known to play a role in the etiology of a number of illnesses due to their dual roles as immunological modulators and phagocytic cells [15,20]. Lymphocytes are essential for adaptive immunity, which helps fight infections [21]. Since these three immune cells play crucial roles in sepsis, SIRI provides a comprehensive representation of immune suppression and inflammation, two characteristics essential to the pathophysiology of sepsis [15,17,18].

Recent studies have assessed both SIRI and SOFA scores in sepsis patients [15,18,22]. Hence, we conducted this study in our hospital to evaluate and compare the SIRI of male and female patients with sepsis. We also correlated the study subjects' SIRI and SOFA scores during admission.

## Materials and methods

We conducted this retrospective study from August 2024 to July 2025 at Kalinga Institute of Medical Sciences (KIMS) in Bhubaneswar, India. The Institutional Ethics Committee provided ethics approval for this study (KIIT/KIMS/IEC/2279/2025, dated 18.08.2025).

Study criteria

We included adult patients of both sexes admitted to our hospital during the study period who fulfilled Sepsis-3 criteria [23]. We excluded patients with any malignancy, immunocompromised state, ongoing chemotherapy, steroids, or blood component transfusion. We also excluded the patients referred from other hospitals and those who died within or stayed for less than seven days.

Study procedure

We recorded the sociodemographic data (i.e., age, sex, socioeconomic status, and marital status) from the subjects' discharge summaries. We used the Kuppuswamy classification to classify the socioeconomic class [24]. We noted the following clinical parameters upon admission to the hospital: total leukocyte count (TLC), ANC, ALC, AMC, and SOFA score. At days 3 and 7, the SOFA scores were noted again. The normal ranges of TLC, ANC, ALC, and AMC are 4.0-11.0 x 10^9^ cells/L, 1.5-8.0 x 10^9^ cells/L, 1.0-4.8 x 10^9^ cells/L, and 0.2-0.8 x 10^9^ cells/L, respectively. We computed SIRI values from the collected ANC, ALC, and AMC data. SIRI is calculated using the following formula: ANC × AMC/ALC (unit: 10^9^/L) [15-18]. We assessed the correlation between baseline SOFA score and SIRI values of the subjects. We also performed the subgroup analysis of the correlation based on the outcome (i.e., death or discharge), difference in SOFA score at day 7 (<2 or ≥2), vasopressor (required or not), multiorgan dysfunction syndrome (MODS) (present or not), age group (≤65 or >65 years), and duration of hospitalization (>14 or ≤14 days).

Statistical analysis

For this study, we used a non-probability consecutive sampling. The Shapiro-Wilk test was used to assess the normality of data distribution. The continuous variables were reported as medians and interquartile ranges (IQRs). Categorical variables were presented as numbers and percentages. The categorical and continuous variables were analyzed with the chi-square and the Wilcoxon test, respectively. We used the Spearman correlation to assess the association between baseline SOFA scores and SIRI in the subjects. We reported the coefficients of correlation with 95% confidence intervals (CIs). For data analysis, we used version 4.3.2 of the R software (R Foundation for Statistical Computing, Vienna, Austria) [25]. A p-value ≤ 0.05 was considered statistically significant.

## Results

During the study period, 3695 patients were admitted to the medicine ward. Nine hundred seventy-six patients either died or left the hospital against medical advice within seven days of their admission. Seven hundred eleven did not develop sepsis. The data of the remaining 2008 patients were analyzed in this study. Tables 1, 2 show the sociodemographic and clinical details of the 2008 patients, respectively. The median age of the study population was 61.0 (53.0-68.0) years. There was a male preponderance (1170, 58.27%) in the study population. The median hospitalization duration was 11.0 (9.0-15.0) days. Around 1569 (78.14%) subjects had MODS. Five hundred fifteen (25.65%) subjects were hospitalized for > 14 days, and 989 (49.25%) patients were on vasopressors. The median SOFA score during admission was 4.0 (2.0-7.0).

**Table 1:** Sociodemographic details of the study population The categorical variables were presented as numbers and percentages, and assessed with the chi-square test. The continuous variables were presented as median and IQR, and assessed with the Wilcoxon test. The test statistics for categorical and continuous variables were the chi-square value and t-value, respectively. The statistical significance is set at p < 0.05. IQR: interquartile range

Parameters	Total (n = 2008)	Female (n = 838)	Male (n = 1170)	Statistical test used	Test statistics	p-value
Age (years)	61.00 (53.00-68.00)	60.00 (53.00-65.00)	62.00 (54.00-70.00)	Wilcoxon test	8.060	< 0.001
Elderly (Age > 65 years)	644 (32.07%)	194 (23.15%)	450 (38.46%)	Chi-square test	35.172	< 0.001
Marital status						
Married	1798 (89.54%)	743 (88.66%)	1055 (90.17%)	Chi-square test	74.134	< 0.001
Unmarried	124 (6.18%)	43 (5.13%)	81 (6.92%)			
Divorced/widowed	86 (4.28%)	52 (6.21%)	34 (2.91%)			
Socioeconomic status						
Low	1211 (60.31%)	504 (60.14%)	707 (60.43%)	Chi-square test	71.688	< 0.001
Lower middle	721 (35.91%)	305 (36.40%)	416 (35.55%)			
Upper middle	76 (3.78%)	29 (3.46%)	47 (4.02%)			

**Table 2:** Clinical traits of the study population The categorical variables were presented as numbers and percentages, and assessed with the chi-square test. The continuous variables were presented as median and IQR, and assessed with the Wilcoxon test. The SOFA scores were assessed with the SOFA scoring system [11]. The test statistics for categorical and continuous variables were the chi-square value and t-value, respectively. The statistical significance is set at p < 0.05. The normal ranges of TLC, ANC, ALC, and AMC are 4.0-11.0 x 10^9^ cells/L, 1.5-8.0 x 10^9^ cells/L, 1.0-4.8 x 10^9^ cells/L, and 0.2-0.8 x 10^9^ cells/L, respectively. IQR: interquartile range; SIRI: Systemic Inflammatory Response Index; SOFA: Sequential Organ Failure Assessment Score; MODS: multiorgan dysfunction syndrome; TLC: total leukocyte count; ANC: absolute neutrophil count; ALC: absolute lymphocyte count; AMC: absolute monocyte count

Parameters	Total (n = 2008)	Female (n = 838)	Male (n = 1170)	Statistical test used	Test statistics	p-value
Total leukocyte count (10^9^/L)	14.72 (11.96-18.49)	14.87 (11.99-18.26)	14.64 (11.91-18.78)	Wilcoxon test	0.051	0.780
Absolute neutrophil count (10^9^/L)	12.56 (10.28-15.60)	12.73 (10.41-15.28)	12.42 (10.22-15.95)	Wilcoxon test	0.067	0.620
Absolute lymphocyte count (10^9^/L)	1.23 (0.81-1.87)	1.16 (0.79-1.83)	1.29 (0.83-1.91)	Wilcoxon test	3.248	0.024
Absolute monocyte count (10^9^/L)	0.54 (0.37-0.77)	0.49 (0.33-0.71)	0.59 (0.40-0.80)	Wilcoxon test	11.040	< 0.001
SIRI (10^9^/L)	5.41 (4.14-6.59)	5.11 (4.03-6.29)	5.55 (4.26-6.88)	Wilcoxon test	7.289	< 0.001
Hospitalization (days)	11.0 (9.0-15.0)	11.0 (9.0-13.0)	12.0 (9.0-16.0)	Wilcoxon test	0.057	0.709
SOFA score at day 1	4.0 (2.0-7.0)	4.0 (2.0-6.0)	5.0 (3.0-8.0)	Wilcoxon test	0.409	0.117
SOFA score at day 3	5.0 (2.0-8.0)	5.0 (2.0-8.0)	5.0 (2.0-8.0)	Wilcoxon test	0.027	0.818
SOFA score at day 7	4.0 (1.0-8.0)	4.0 (1.0-8.0)	5.0 (1.3-8.0)	Wilcoxon test	1.375	0.011
Difference in SOFA scores	0.0 (-2.0-2.0)	0.0 (-2.0-2.0)	-1.0 (-2.0-1.8)	Wilcoxon test	3.019	< 0.001
MODS	1569 (78.14%)	619 (73.87%)	950 (81.20%)	Chi-square test	6.713	< 0.001
Vasopressor required	989 (49.25%)	425 (50.72%)	564 (48.21%)	Chi-square test	10.128	< 0.001
Long duration of stay (hospitalization > 14 days)	515 (25.65%)	148 (17.66%)	367 (31.37%)	Chi-square test	14.047	< 0.001
Outcome						
Discharge	1741 (86.70%)	728 (86.87%)	1013 (86.58%)	Chi-square test	77.528	< 0.001
Death	267 (13.30%)	110 (13.13%)	157 (13.42%)			

**Figure 1:**
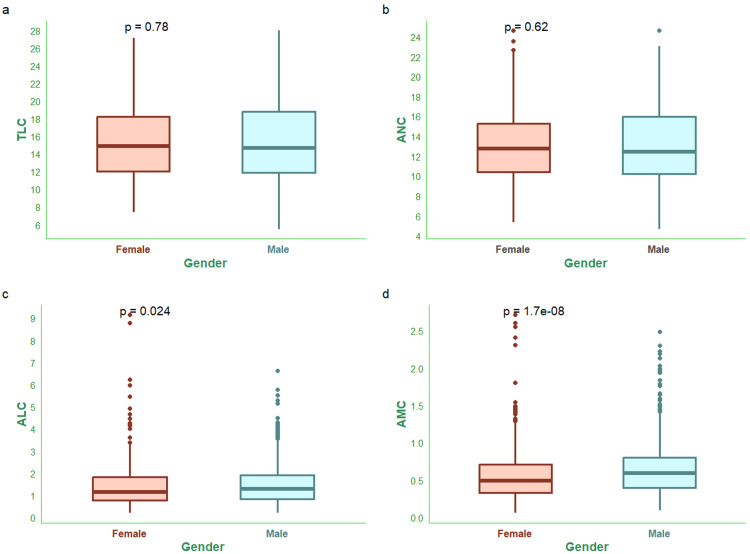
TLC, ANC, ALC, and AMC of the study population The box-and-whisker plots in panels a, b, c, and d show TLC (x 10^9^/L), ANC (x 10^9^/L), ALC (x 10^9^/L), and AMC (x 10^9^/L) of female and male participants, respectively. The normal ranges of TLC, ANC, ALC, and AMC are 4.0-11.0 x 10^9^ cells/L, 1.5-8.0 x 10^9^ cells/L, 1.0-4.8 x 10^9^ cells/L, and 0.2-0.8 x 10^9^ cells/L, respectively. The intergroup comparisons were performed with the Wilcoxon test. The statistical significance is set at p < 0.05. TLC: total leukocyte count; ANC: absolute neutrophil count; ALC: absolute lymphocyte count; AMC: absolute monocyte count

Figure 1 shows TLC, ANC, ALC, and AMC of the study population. The median TLCs of female and male subjects were 14.87 (11.99-18.26) x 10^9^/L and 14.64 (11.91-18.78) x 10^9^/L (Figure 1a). The median ANCs of female and male subjects were 12.73 (10.41-15.28) x 10^9^/L and 12.42 (10.22-15.95) x 10^9^/L (Figure 1b). The median ALCs of female and male subjects were 1.16 (0.79-1.83) x 10^9^/L and 1.29 (0.83-1.91) x 10^9^/L (Figure 1c). The median AMCs of female and male subjects were 0.49 (0.33-0.71) x 10^9^/L and 0.59 (0.40-0.80) x 10^9^/L (Figure 1d).

**Figure 2:**
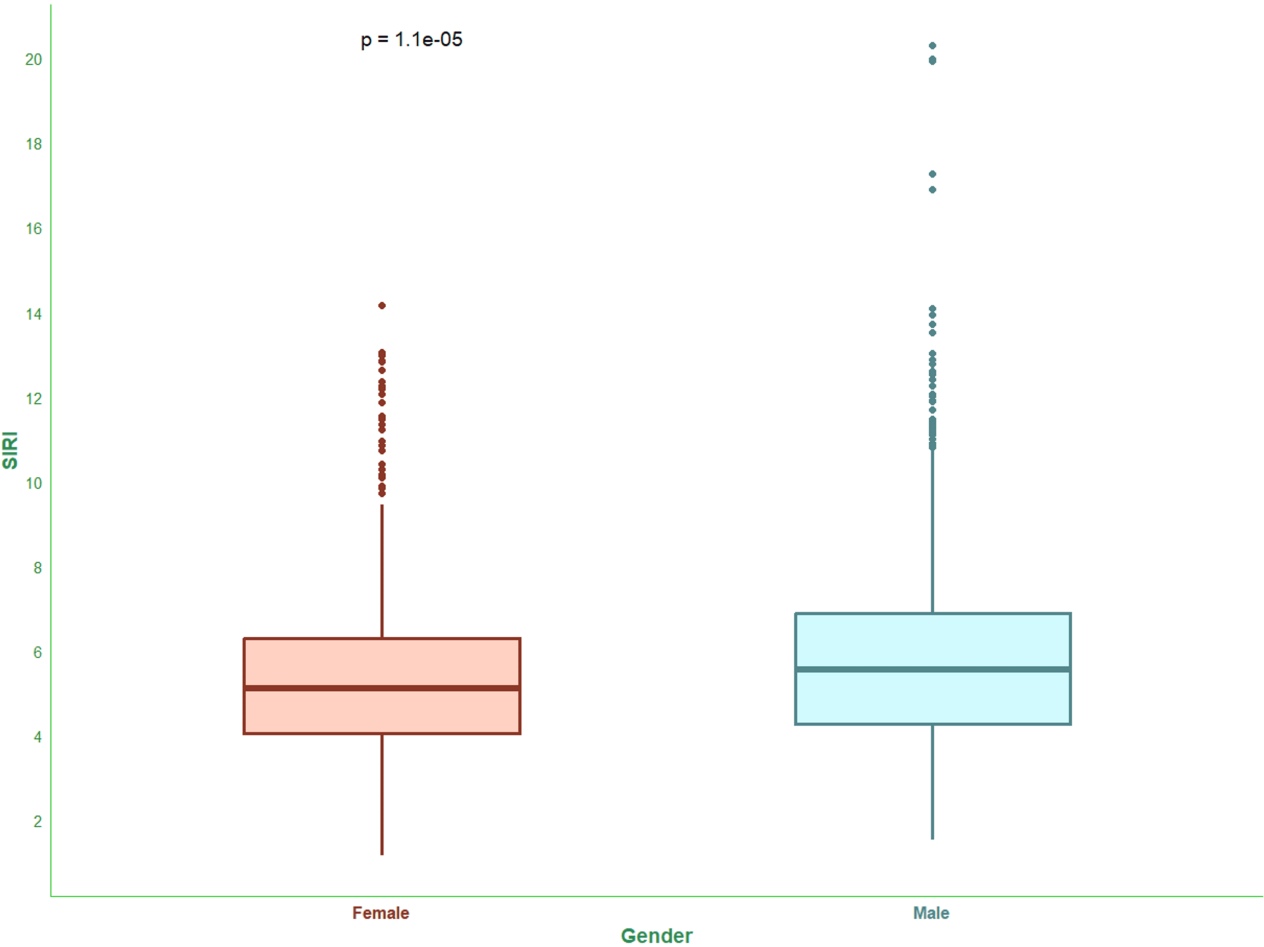
SIRI values of the study population The box-and-whisker plots show SIRI values (×10^9^/L) for female and male participants. The statistical significance is set at p < 0.05. The intergroup comparisons were performed with the Wilcoxon test. SIRI: Systemic Inflammatory Response Index

Figure 2 shows SIRI values of the study population. The median SIRI values of female and male subjects were 5.11 (4.03-6.29) x 10^9^/L and 5.55 (4.26-6.88) x 10^9^/L.

**Figure 3:**
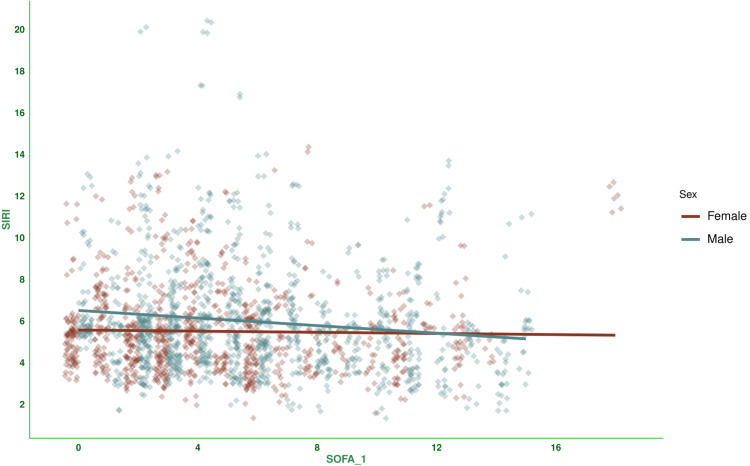
Correlation between SIRI and SOFA scores of female and male participants The jitter plots show the correlation between baseline SOFA scores and SIRI values in the study population. The SOFA scores were assessed with the SOFA scoring system [11]. The Spearman correlation was used to check the association. The statistical significance is set at p < 0.05. SIRI: Systemic Inflammatory Response Index, SOFA: Sequential Organ Failure Assessment Score, SOFA_1: SOFA score at day 1

**Figure 4:**
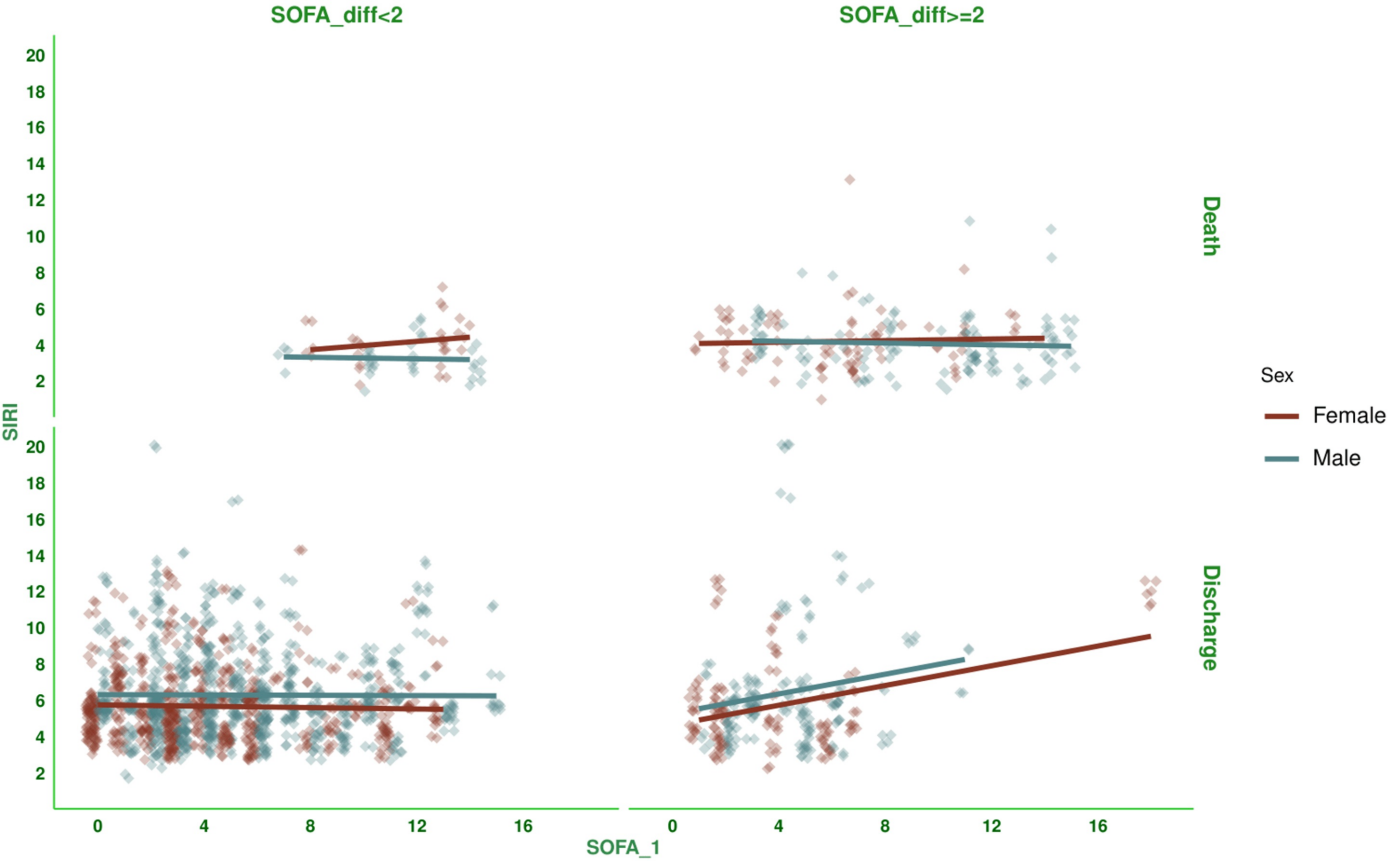
Correlation between SIRI and SOFA score based on outcome and the difference in SOFA scores of the study population The jitter plots show the correlation between baseline SOFA scores and SIRI values in the study population. The horizontal and vertical grids present the outcome (i.e., death or discharge) and difference in SOFA score at day 7 from that at day 1 (<2 or ≥2), respectively. The SOFA scores were assessed with the SOFA scoring system [11]. The Spearman correlation was used to analyze the association. The statistical significance is set at p < 0.05. SIRI: Systemic Inflammatory Response Index; SOFA: Sequential Organ Failure Assessment Score; SOFA_1: SOFA score at day 1; SOFA_diff: difference in SOFA score at day 7 from that at day 1

**Figure 5:**
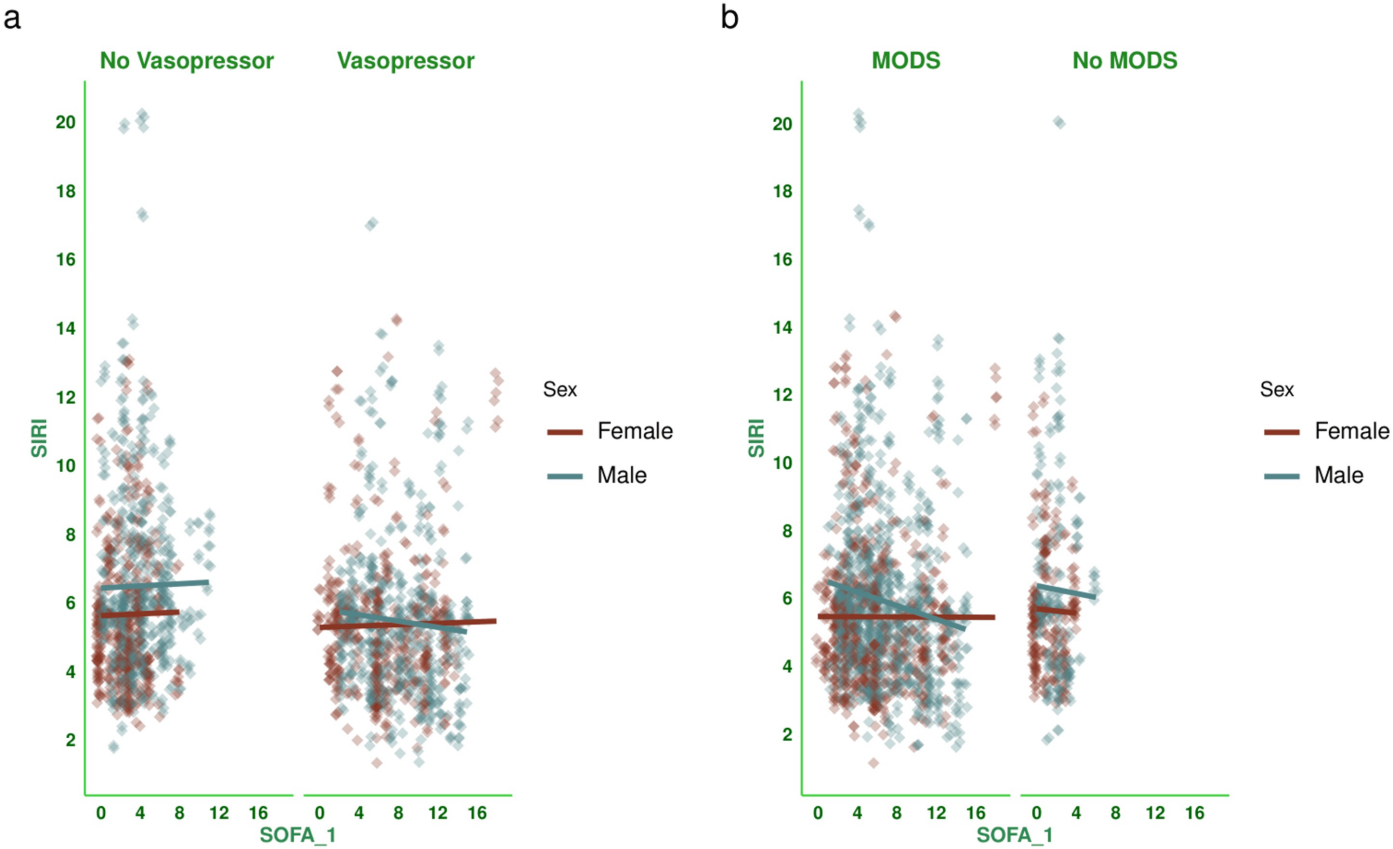
Correlation between SIRI and SOFA score based on the requirement of vasopressors and presence of MODS The jitter plots show the correlation between baseline SOFA scores and SIRI values of the study population. Panels a and b show the subgroup analyses based on the requirement for vasopressors and the presence of MODS, respectively. The SOFA scores were assessed with the SOFA scoring system [11]. The Spearman correlation was used to check the association. The statistical significance is set at p < 0.05. SIRI: Systemic Inflammatory Response Index; SOFA: Sequential Organ Failure Assessment Score; SOFA_1: SOFA score at day 1; MODS: multiorgan dysfunction syndrome

**Figure 6:**
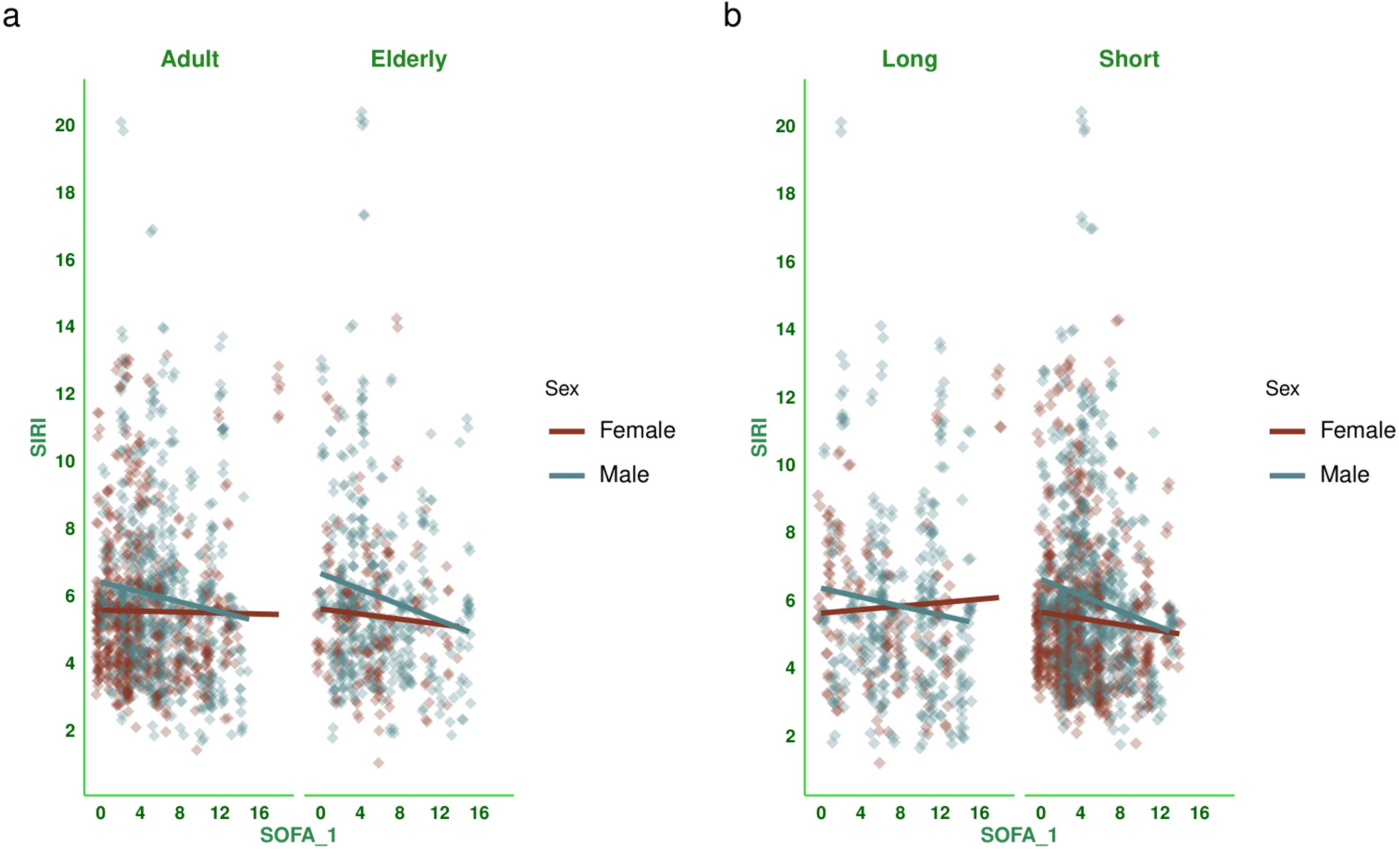
Correlation between SIRI and SOFA score based on the age group and duration of hospitalization The jitter plots show the correlation between baseline SOFA scores and SIRI values of the study population. Panels a and b show the subgroup analyses by age group and hospitalization duration, respectively. The individuals of age ≤65 years and >65 years were regarded as adults and the elderly, respectively. The duration of hospitalization of >14 days and ≤14 days was regarded as long and short duration, respectively. The SOFA scores were assessed with the SOFA scoring system [11]. The Spearman correlation was used to check the association. The statistical significance is set at p < 0.05. SIRI: Systemic Inflammatory Response Index; SOFA: Sequential Organ Failure Assessment Score; SOFA_1: SOFA score at day 1

Figure 3 shows the correlation between baseline SOFA scores and SIRI values for the subjects. There was a negative correlation between the parameters, with a coefficient of -0.078 (-0.121 to -0.034, p < 0.001). Figure 4 shows the subgroup analysis of this correlation between baseline SOFA score and SIRI based on the outcome (i.e., death or discharge) and difference in SOFA score at day 7 (<2 or ≥2). The majority of correlations were either weakly positive or weakly negative. Participants who were discharged and had a difference in SOFA score ≥2 were the exception (0.256, 95% CI: 0.147 to 0.359, p < 0.001). Figure 5 shows the subgroup analysis of the correlation between baseline SOFA score and SIRI, stratified by vasopressor use (yes/no) and MODS (present/absent). There were mostly weakly negative correlations between SIRI and SOFA score (subjects on vasopressors: (-0.028, 95% CI: -0.090 to 0.035, p = 0.386), subjects with MODS: (-0.077, 95% CI: -0.126 to -0.028, p = 0.002), and subjects without MODS: (-0.019, 95% CI: -0.112 to 0.075, p = 0.690)). Figure 6 shows the subgroup analysis of the correlation between baseline SOFA score and SIRI by age group (≤65 or >65 years) and hospitalization duration (< 14 or> 14 days). There were negative correlations between SIRI and SOFA score (adult subjects (i.e., ≤65 years): (-0.051, 95% CI: -0.104 to 0.002, p = 0.061), elderly subjects (i.e., >65 years): (-0.132, 95% CI: -0.208 to -0.056, p < 0.001), subjects with long hospital stay (i.e., >14 days): (-0.054, 95% CI: -0.140 to 0.033, p = 0.222), and subjects with short hospital stay (i.e., ≤14 days): (-0.103, 95% CI: -0.153 to -0.053, p < 0.001)). Table 3 presents all correlation coefficients, their 95% CIs, and p-values.

**Table 3:** Correlation between SIRI and SOFA scores of the study population The Spearman correlation was used to test the association. The total population was noted as "Total" in the first row. The individuals of age ≤65 years and >65 years were regarded as adults and the elderly, respectively. The duration of hospitalization of >14 days and ≤14 days was regarded as long and short duration, respectively. The SOFA scores were assessed with the SOFA scoring system [11]. The statistical significance is set at p < 0.05. SIRI: Systemic Inflammatory Response Index; SOFA: Sequential Organ Failure Assessment Score; MODS: multiorgan dysfunction syndrome; SOFA_diff: difference in SOFA score at day 7 from SOFA score at day 1

Parameters	Total (n = 2008)		Female (n = 838)		Male (n = 1170)	
	r (95% CI)	p-value	r (95% CI)	p-value	r (95% CI)	p-value
Total	-0.078 (-0.121 to -0.034)	< 0.001	-0.024 (-0.092 to 0.044)	0.485	-0.130 (-0.186 to -0.073)	< 0.001
SOFA_diff <2 and death	0.098 (-0.147 to 0.332)	0.432	0.203 (-0.170 to 0.525)	0.283	-0.049 (-0.371 to 0.284)	0.777
SOFA_diff ≥2 and death	-0.033 (-0.171 to 0.106)	0.641	0.045 (-0.176 to 0.263)	0.689	-0.060 (-0.236 to 0.120)	0.513
SOFA_diff <2 and discharge	-0.001 (-0.052 to 0.051)	0.974	-0.032 (-0.112 to 0.048)	0.429	-0.006 (-0.074 to 0.061)	0.852
SOFA_diff ≥2 and discharge	0.256 (0.147 to 0.359)	< 0.001	0.381 (0.221 to 0.520)	< 0.001	0.172 (0.023 to 0.313)	0.024
Vasopressor	-0.028 (-0.090 to 0.035)	0.386	0.019 (-0.076 to 0.114)	0.692	-0.129 (-0.271 to 0.019)	0.088
No vasopressor	0.042 (-0.019 to 0.104)	0.176	0.012 (-0.085 to 0.108)	0.807	0.014 (-0.066 to 0.094)	0.731
MODS	-0.077 (-0.126 to -0.028)	0.002	-0.002 (-0.081 to 0.077)	0.957	-0.137 (-0.198 to -0.074)	< 0.001
No MODS	-0.019 (-0.112 to 0.075)	0.690	-0.024 (-0.156 to 0.109)	0.721	-0.028 (-0.160 to 0.105)	0.681
Adult	-0.051 (-0.104 to 0.002)	0.061	-0.013 (-0.090 to 0.065)	0.747	-0.108 (-0.180 to -0.035)	0.004
Elderly	-0.132 (-0.208 to -0.056)	< 0.001	-0.024 (-0.156 to 0.109)	0.721	-0.028 (-0.160 to 0.105)	0.681
Long duration	-0.054 (-0.140 to 0.033)	0.222	0.054 (-0.109 to 0.213)	0.518	-0.100 (-0.200 to 0.003)	0.056
Short duration	-0.103 (-0.153 to -0.053)	< 0.001	-0.073 (-0.147 to 0.002)	0.056	-0.140 (-0.207 to -0.071)	< 0.001

## Discussion

In this retrospective study, 2008 patients were assessed. Their median age was 61.0 (53.0-68.0) years. There were 1170 (58.27%) male subjects. The median hospitalization duration was 11.0 (9.0-15.0) days. Around 1569 (78.14%) subjects had MODS. Five hundred fifteen (25.65%) subjects were hospitalized for > 14 days, and 989 (49.25%) patients were on vasopressors. The median SOFA score during admission was 4.0 (2.0-7.0). There was a negative correlation between SOFA score at day 1 and SIRI (-0.078, 95% CI: -0.121 to -0.034, p < 0.001). The majority of the subgroup analyses yielded similar findings. Our observations regarding SIRI matched those of Zhu et al. [15] and Ru et al. [22].

Globally, the SOFA score is used to measure the morbidity and prognosis of critically ill patients. But its computation requires multiple factors and routine observations [14,26]. CRP, procalcitonin, interferons, adrenomedullin, IL-6, and ICAM-1 are some of the recent biomarkers for sepsis [12,13]. The higher cost and lesser availability of these biomarkers limit their usage [12,14].

Another marker of inflammatory, immune-mediated sepsis and septic shock is the Systemic Immune-Inflammation Index (SII) [27]. SIRI has been identified as a predictive marker for sepsis [15,17,18]. By establishing a substantial association between SIRI and sepsis, this study demonstrates SIRI's predictive value in critically ill patients [15,28]. This indicator evaluates systemic inflammation and immunological balance in real time. The degree of inflammation is greatly influenced by neutrophils, which are crucial mediators of innate immunity, due to their active state [19]. Monocytes' dual roles as phagocytic clearance and immunological modulators have led to their involvement in the etiology of several diseases [15,20]. Lymphocytes play a critical role in understanding the high morbidity and long-term mortality linked to sepsis [21,28]. SIRI takes lymphocytes into account. Hence, it might offer a more thorough evaluation of the inflammatory response in individuals with sepsis [15,21,28].

Strengths and limitations

Our study was strengthened by the evaluation and correlation of SIRI and SOFA scores among the study subjects. Our study had a few limitations. First, a single-centered study and a small sample size limit the generalizability of the findings. Second, missing data on renal, hepatic, hemodynamic, and glycemic parameters; comorbidities; concomitant medications; antibiotics used; urine output; and daily fluid intake led us to exclude some patients. Third, referred patients were also excluded because their indices could have been miscalculated due to previous interventions.

## Conclusions

The higher TLC and ANC values of the study population than the normal range and the normal ALC and AMC values were suggestive of either a recent infection or an ongoing infection with non-compromised immune status. Male and female participants showed similar hematological parameters. However, SIRI and SOFA scores were higher in male subjects. As the associations between SIRI and SOFA scores were weak, our study findings cannot be generalized. Our study was a single-center retrospective study. Hence, we suggest prospective, multicenter studies with larger sample sizes and longer follow-up to explore the potential of SIRI as a biomarker for sepsis.
